# Changes in Non-Nutritive Sweetener Consumption Patterns in Response to a Sugar-Sweetened Beverage Reduction Intervention

**DOI:** 10.3390/nu12113428

**Published:** 2020-11-08

**Authors:** Darlene Acero, Jamie M. Zoellner, Brenda M. Davy, Valisa E. Hedrick

**Affiliations:** 1 Department of Human Nutrition, Foods, and Exercise, Virginia Tech, 295 West Campus Drive, Blacksburg, VA 24061, USA; dacero@vt.edu (D.A.); bdavy@vt.edu (B.M.D.); 2 Department of Public Health Sciences, Cancer Center without Walls, University of Virginia, 16 East Main St., Christiansburg, VA 24073, USA; jz9q@virginia.edu

**Keywords:** non-nutritive sweeteners, artificial sweeteners, added sugars, sugar-sweetened beverage intervention

## Abstract

Data are lacking on whether non-nutritive sweeteners (NNS) can be used as a strategy to support decreases in sugar-sweetened beverage (SSB) consumption. The purpose of this secondary analysis of a 6-month SSB-reduction intervention was to explore changes in NNS consumption patterns in Talking Health participants within the SIPsmartER (*n* = 101) intervention. Additionally, participant characteristics were compared for three SSB-NNS change groups (decrease SSB/increase NNS; decrease SSB/no increase in NNS; increase/no change in SSB/regardless of NNS). There was a significant increase in aspartame and total NNS intake for participants (mean daily mg increases of 37.2 ± 13.9 and 63.7 ± 18.5, respectively). With the exception of sex, no differences in participant characteristics were found between the three SSB-NNS change groups. Furthermore, no significant changes in weight or body mass index (BMI) were demonstrated between SSB-NNS change groups over time. Diet soda was the most commonly consumed source of NNS; however, other dietary sources of NNS also contributed to intake. At 6 months, intake of sucralose and saccharin were primarily from dietary sources other than diet sodas (94% and 100%, respectively). These findings suggest that NNS may be a feasible strategy to help reduce SSB consumption. This study supports the need to consistently quantify and identify NNS intake, beyond using diet soda intake as a proxy for NNS intake and grouping all NNS types into one variable, to more accurately address the potential health effects of NNS.

## 1. Introduction

The overconsumption of added sugars in the United States is widespread, with only 42% of Americans, 2 years of age and older, meeting the recommendation of less than 10% of daily calories from added sugars set by the 2015–2020 Dietary Guidelines for Americans [1]. In addition to its relationship with obesity, excessive consumption of added sugars is linked to multiple health outcomes such as diabetes, non-alcoholic fatty liver disease, and some forms of cancer [2]. Due to the adverse effects of added sugars on health and their widespread consumption, public health advocates have searched for methods to reduce consumption. One suggested method is the replacement of added sugars in food and beverage products with non-nutritive sweeteners (NNS), more commonly known as artificial sweeteners. 

Approximately half (40−50%) of added sugars in the American diet can be attributed to the consumption of sugar-sweetened beverages (SSB) [3]. NNS are commonly used in diet sodas as substitutes for added sugars [4]. Because of this, consumers look to beverages sweetened with NNS as a substitute for SSB. In addition to their popular use in diet sodas, NNS are used in diet teas, juice drinks, tabletop sweeteners, desserts, chewing gum, and other items such as toothpaste [4,5]. Currently, the types of NNS approved as food additives by the United States Food and Drug Administration include acesulfame potassium, aspartame, saccharin, sucralose, neotame, and advantame [5]. Stevioside, rebaudioside A, and luo gan guo (monkfruit) are not approved as food additives, but rather are designated as “generally recognized as safe” (GRAS) [4].

Despite consumption of NNS increasing over time [6], research on the health benefits and/or detriments of NNS consumption is limited, and results from research on the effects of NNS consumption are varied. Reported associations include weight loss [7,8,9,10,11,12,13] and improved fasting serum insulin and 2-h postprandial glucose concentrations [9,14], and conversely, weight gain [7,10,15,16,17] and increased fasting plasma glucose concentrations [18,19]. The inability to reach a consensus of the impact of NNS consumption on health may be attributed to limitations of existing research. Some of these limitations include the use of observational studies, rather than randomized controlled trials on humans, which cannot establish causality [7,10,15,16,17,18,20,21,22], randomized controlled trials limited to rodents [19,23,24,25,26], the inclusion of weight loss interventions [8,9,11,12,14], the use of diet soda as a proxy for NNS consumption [9,11,13,14,18,22,27,28], and the study of NNS as a whole rather than by specific type [9,11,12,13,14,15,16,18,22,27,29]. It is necessary to differentiate between the types of NNS, since not all types are metabolically the same, and thus may have different effects on health. Data are lacking on whether NNS consumption is a potential tool to help decrease SSB consumption. If NNS consumption is used as a means to reduce SSB intake, it is important to demonstrate the changes in the sources and types of NNS that may occur. The data regarding changes in NNS intake can help inform future SSB reduction trials by providing specific demographic groups with targeted intervention content regarding NNS trends, strategies, and potential barriers. To improve NNS research and its related effects on health, it is important to address limitations of the current research.

The main purpose of this secondary analysis of Talking Health trial data [30,31] was to characterize changes in NNS consumption (frequency of NNS consumers to non-consumers, consumption of total NNS and by specific types, and dietary sources of NNS) in response to a 6-month SSB reduction intervention (SIPsmartER). A secondary objective was to characterize the demographic characteristics of SIPsmartER participants who chose to use NNS as a tool to reduce SSB intake relative to those who did not.

## 2. Materials and Methods 

### 2.1. Study Design and Subjects

This secondary analysis utilized data from Talking Health, a type 1 effectiveness-implementation hybrid randomized-controlled behavioral trial targeting community residents in rural southwest Virginia [30]. The primary objective of Talking Health was to examine the effectiveness of a 6-month intervention trial aimed at decreasing SSB intake (SIPsmartER) when compared to a matched-contact group targeting physical activity (MoveMore) [31]. In the original investigation, both SIPsmartER and MoveMore groups significantly decreased their SSB intake over the six months, but SIPsmartER participants decreased SSB significantly more as compared to MoveMore participants [30].

Talking Health targeted residents in an 8-county rural region of southwest Virginia [31]. Using the Rural-Urban Continuum Codes (1 = urban, 9 = completely rural), the targeted counties had an average rurality status of 6.1 ± 2.5 (with a range of 2 to 9) [32]. The location of this study, southwest Virginia, is of importance as low socioeconomic adults in rural areas have a higher probability of consuming more SSB [30]. Eligible participants for Talking Health were English-speaking adults ≥18 years of age, who consumed ≥ 200 SSB kcals/day, with no self-reported contraindications for physical activity, and with regular access to a telephone. Although not part of the inclusion criteria, recruitment efforts targeted low socioeconomic individuals and those with limited health literacy.

Data were collected at baseline and 6 months. Participants were either randomized into SIPsmartER (*n* = 155) or MoveMore (*n* = 146) at baseline. The goal of SIPsmartER was to decrease SSB consumption to the recommended level of ≤8 fluid ounces per day, while the goal of MoveMore was to increase physical activity to the recommended amount of 150 min of moderate-intensity and/or muscle strengthening activities per week. Over the 6-month period, both interventions consisted of 3 small group classes, 1 live teach-back phone call, and 11 interactive voice response phone calls [31]. During 1 of the 3 small group classes, SIPsmartER participants received information on the negative health effects of SSB overconsumption and potential replacements such as water and beverages sweetened with NNS. The controversy surrounding NNS consumption was addressed, and consumption of NNS was presented as a personal choice and a healthy and safe alternative to SSB if consumers wished to consume them. Participants were given information regarding all beverage types, in addition to water, SSB, and NNS beverages, including milk, juice, coffee, tea, and alcohol. 

For this secondary analysis, only participants within the SIPsmartER intervention were included. Participants who did not return at 6 months (*n* = 41), were pregnant at either baseline or 6 months (*n* = 4), or were considered an outlier (more than 3 standard deviations from the mean for total NNS consumption) at either baseline or 6 months (*n* = 6) were not included. Additionally, 3 participants were removed due to incomplete dietary recall data. A final sample for SIPsmartER (*n* = 101) was calculated. This trial was registered at Clinicaltrials.gov; ID: NCT02193009.

### 2.2. Methods

Demographic characteristics (age, sex, ethnicity/race, income, education status) were assessed at baseline [31]. At baseline and 6 months, anthropometrics (height, weight, and body mass index (BMI)) and three 24-h dietary recalls were collected. Weight was measured at both time points with light clothing and without shoes using a calibrated digital Tanita scale (Model: 310 GS, Tanita Corporation, Arlington Heights, IL, USA), and height was measured only at baseline via a research-grade stadiometer.

Dietary intake was collected by trained researchers, supervised by a PhD-level Registered Dietitian Nutritionist, via three non-consecutive 24-h dietary recalls within a two-week period, capturing two weekdays and one weekend day. Interviewer-administered methods were used to collect dietary recalls, with one day completed in-person and the other two days completed via unannounced telephone calls. Nutritional analysis software (Nutrition Data System for Research (NDS-R) 2011, Nutrition Coordinating Center, University of Minnesota, Minneapolis, MN, USA) was used to analyze the 24-h dietary recalls [31].

### 2.3. NNS Consumption Extraction 

Baseline and 6-month 24-h dietary recalls were analyzed via NDS-R to characterize frequency of NNS consumers to non-consumers and to examine intake of specific types of NNS, total NNS, and dietary sources of NNS [31]. The milligrams (mg) of NNS by types (aspartame, sucralose, saccharin, and acesulfame potassium) in NNS-containing food and beverage items were then extracted from the component/ingredient level of participants’ dietary intake [33]. Categorization of NNS consumers and non-consumers were determined by the mean total mg of NNS consumed in one day from both foods and beverages in a participant’s diet. A participant was considered a NNS consumer if they consumed the equivalent of NNS found in 1 fl oz of diet soda from either foods or beverages [5]. This corresponds to 3 mg acesulfame potassium, 17 mg aspartame, 12 mg saccharin, or 6 mg sucralose. This categorization ensured that intentional intake occurred and NNS sources other than diet sodas were accounted for when determining NNS consumer status.

#### 2.3.1. Analysis of NNS Consumption Patterns 

Participants’ NNS consumption patterns were examined by looking at changes over time in proportions of NNS consumers to non-consumers and changes in consumption of specific NNS types (aspartame, saccharin, acesulfame potassium, sucralose) and total NNS. Average daily NNS intake by mg content was determined for specific types and total NNS. Using only NNS consumers (at baseline, 6 months, or both), changes over the 6-month intervention in the most commonly consumed NNS types were identified, along with dietary sources of NNS (e.g., diet soda, tabletop sweeteners, etc.). Dietary sources of NNS at both baseline and 6 months were analyzed by total mg intake.

#### 2.3.2. Analysis of NNS Consumer Demographics

Descriptive consumption data were used to examine the continuum and magnitude of changes (none, small, medium, large) in both SSB and NNS consumption using all SIPsmartER participants. Similar groups were combined to create larger and more comparable groups for a total of three groups. They were categorized by amount of change and are as follows:

- Group 1 (*n* = 36): decreased SSB consumption (≥1.5 fl oz) with increased NNS consumption (>3.0 total NNS mg)
- Group 2 (*n* = 43): decreased SSB consumption (≥1.5 fl oz) but no increase in NNS consumption (≤2.99 total NNS mg)
- Group 3 (*n* = 22): increased/no change in SSB consumption, regardless of NNS consumption

An intake level of at least 3 mg of total NNS was determined as the cutoff for “increased” intake in group 1 to match the definition of an NNS consumer stated above (the equivalent of NNS in 1 fl oz of diet soda, which corresponds to 3 mg acesulfame potassium, 17 mg of aspartame, 12 mg of saccharin, or 6 mg of sucralose).

Demographic profiles (sex, age, race/ethnicity, weight, BMI, BMI category, educational level, household income, and mean income) between these three groups were compared at 6 months, along with changes in SSB consumption (fl oz), total NNS intake (mg), weight (kg), and BMI (kg/m^2^) over the 6-month intervention.

### 2.4. Statistical Analyses 

Statistical analyses were performed using SPSS statistical software (version 25.0 for Macintosh, 2017; IBM Corporation, Armonk, NY, USA). To aid in statistical interpretation, exact *p*-values were reported for main outcomes, and *p*-value inequalities (*p* ≤ 0.05, *p* ≤ 0.01, and *p* ≤ 0.001) were reported for other secondary comparisons.

Changes in NNS intake (specific types and total) were analyzed via descriptive statistics (mean ± standard deviation, frequencies) and paired samples *t*-tests. Using only NNS consumers (baseline, 6 months, or both), descriptive statistics were utilized to determine mg intake and intake per dietary source for each type of NNS (acesulfame potassium, aspartame, saccharin, sucralose) and total NNS.

The groups analyzed for the secondary aim were the three SSB-NNS consumption change groups defined previously: (1) decreased SSB consumption with increased NNS consumption, (2) decreased SSB consumption with no change in NNS consumption, and (3) increased/no change in SSB consumption regardless of NSS consumption). Demographics were analyzed using descriptive statistics (mean ± standard deviation and frequencies). One-factor ANOVAs with Tukey’s post hoc tests were used to evaluate differences in age, weight, BMI, and mean household income between groups at 6 months. X^2^ tests with adjusted standardized residuals were used to evaluate differences in sex, race/ethnicity, BMI category, education level, and household income category between groups. Descriptive statistics (mean ± standard deviation) were calculated for SSB consumption, total NNS consumption, weight, and BMI. Changes in SSB consumption, total NNS consumption, weight, and BMI were analyzed via paired sample *t*-tests within groups; differences between groups over time were analyzed via repeated measures ANOVAs with Tukey’s post hoc tests.

## 3. Results

### 3.1. Changes in Frequency of NNS Consumers and Non-Consumers Over Time 

Table 1 shows the changes in frequency of NNS consumers to non-consumers in SIPsmartER participants over time. There was a 16% increase in NNS consumers from baseline to 6 months. 

### 3.2. Changes in Non-Nutritive Sweetener Consumption Over Time

Table 2 shows the changes in mean daily NNS consumption in participants by specific types and total NNS over the 6 months. From baseline to 6 months, there was a significant increase in aspartame and total NNS consumption.

### 3.3. Changes in Non-Nutritive Sweetener Consumption Sources Over Time 

Table 3 illustrates the changes in total daily NNS mg intake from specific dietary sources over the 6 months in participants who were NNS consumers at baseline, 6 months, or both. This table expands on Table 2, which presented mean NNS intake, by reporting the total mg consumed from each dietary source by NNS type and total NNS for all participants.

Additional descriptive analyses showed that while diet soda contributed a large proportion of NNS mg consumed at both baseline and 6 months, additional food products not usually included in NNS intake analyses, such as diet tea, juice drinks, tabletop sweeteners, yogurt, meal replacement products, and others, also contributed to total NNS intake. Furthermore, the different types of NNS (aspartame, sucralose, acesulfame potassium, and saccharin) were found in a variety of these products. At 6 months, intake of sucralose and saccharin were primarily from dietary sources other than diet sodas (94% and 100%, respectively), while intake of aspartame and acesulfame potassium from dietary sources other than diet sodas was lower at 10% and 24%, respectively. Additional findings (not shown) demonstrated that 30% (9/30) and 24% (11/46) of NNS consumers at baseline and 6 months, respectively, did not consume any diet sodas.

### 3.4. Differences in Demographics Between SSB-NNS Consumption Change Groups 

There were no significant differences in age, race, weight status, education, and income status between participants who used NNS as a tool to reduce SSB consumption (group 1: decreased SSB, increased NNS), those who did not use NNS to reduce SSB intake (group 2: decreased SSB, with no change in NNS), and those who were unresponsive to the SIPsmartER intervention (group 3: increased or no change in SSB, regardless of change in NNS). The only significant difference in demographic characteristics was the proportion of males to females, with group 3 having significantly fewer males as compared to groups 1 and 2 (Table 4).

Table 5 shows the difference in SSB and NNS consumption, body weight, and BMI between the three SSB-NNS groups over 6 months. There was a significant overall difference between groups for changes in SSB consumption, however, post hoc tests were unable to detect which means differed between groups. Additional findings (not shown) showed that group 2 (decreased SSB, but no increase in NNS consumption) had a significant increase in water intake over the 6 months compared to groups 1 and 3. Of note, there were no significant group differences in weight or BMI over time. 

## 4. Discussion

This is the first investigation, to our knowledge, to explore changes in NNS consumption patterns after a SSB-reduction intervention. This analysis contributes new information to the current body of research investigating NNS by overcoming several limitations in existing literature, including the use of diet soda as a proxy for NNS consumption and the study of NNS as a whole rather than by specific type. These results suggest that NNS are consumed from many foods other than diet sodas. The inclusion of dietary sources of NNS in addition to diet sodas helps ensure intake of NNS is accurately quantified and that NNS consumers are not incorrectly identified as non-consumers. Furthermore, this study focuses on various specific types of NNS. This is important as not all types of NNS are metabolically the same [34], various types of NNS are sometimes found in one product, and the majority of some NNS types are primarily found in dietary sources other than diet sodas. Accounting for different types and sources of NNS creates a more accurate characterization of NNS intake patterns.

Our secondary analysis of SIPsmartER showed that study participants, when presented with NNS as a safe option, may opt for it as a tool to decrease their intake of SSB. At baseline, the proportions of NNS consumers to non-consumers (30% to 70%) mirrored national trends of 33% [35]. Over the 6 months, the proportion of NNS consumers increased by 16%. Additionally, participants significantly increased their consumption of aspartame (increase was equivalent to the amount of aspartame found in one tabletop sweetener packet) and total NNS over the 6 months. Although the increase in aspartame consumption was significant, the research is currently inconclusive on the potential health implications of this increase [8,19,20,21,26]. Studies focusing on NNS consumption usually emphasize total NNS, and few focus on different types of NNS [8,20,24]. This study’s results demonstrated that consumers consume and change their consumption of different types of NNS in varying amounts. These findings support the need to focus on specific types of NNS, rather than total NNS alone, as health and metabolic outcomes may differ based upon the type of NNS consumed [35].

Despite the analysis on dietary sources of NNS being descriptive, it provides valuable information that adds to the current body of literature on NNS consumption patterns. These results show that while at both baseline and 6 months the most commonly consumed dietary source of NNS by mg intake was diet sodas, there were other sources such as diet tea, fruit drinks, tabletop sweeteners, yogurt, popcorn, cereal, coffee creamer substitutes, ice cream, hot cocoa mix, and meal replacement products consumed as well. Consumption of saccharin and sucralose was attributed mostly to items such as tabletop sweeteners and juice or flavored drinks. Of note, 100% of consumption of saccharin at 6 months came from tabletop sweeteners. Although most current studies consider the most commonly consumed dietary source of NNS (diet beverages) [6], these results highlight the importance of accounting for other sources of NNS beyond diet sodas to ensure accurate characterization of NNS consumption. Additionally, our results showed that 30% of NNS consumers at baseline and 24% of NNS consumers at 6 months did not consume NNS from diet soda. Therefore, had we characterized our NNS consumers solely by NNS consumption from diet sodas, as many studies do, nearly 30% of participants would have been incorrectly defined as non-consumers at both baseline and 6 months. Hess et al., corroborates these findings with a different population [20].

Within the three SSB-NNS consumption change groups for the secondary objective, no significant differences in demographics, with the exception of sex, were found for those who successfully decreased SSB while increasing NNS consumption when compared to those who did not increase NNS but decreased SSB or were not successful at decreasing SSB. The main goal of this aim was to see if there were any differences in demographics between people who may use NNS as a tool to decrease SSB consumption versus those who did not. As the demographics of all three groups mirror common NNS consumer demographics: non-Hispanic, white [6], female [27,36], and more likely to be overweight or obese [27,36], it is possible that the original sample’s homogeneity [30], as well as the small sample size of the three groups, led to a lack of significant differences between groups.

Of note is that group 3 at baseline had relatively lower intake of SSB and higher intake of NNS when compared to groups 1 and 2. Additionally, of those participants who decreased their consumption of SSB (groups 1 and 2) half of those participants (group 1) decreased consumption of SSB while increasing consumption of NNS, while the other half (group 2) did not. Group 2, however, did have a significant increase in water intake when compared to groups 1 and 3, indicating that water consumption may produce similar effects as NNS when used as a tool to reduce SSB intake. In general, the 22 participants who did not change SSB (group 3) were high consumers of NNS at baseline. This study suggests the potential NNS could have as a tool to reduce or maintain low consumption of SSB (groups 1 and 3). However, the focus of SIPsmartER was not on the reduction of SSB exclusively via NNS; thus, further studies are needed to study the potential role of NNS in the reduction of SSB consumption. Additionally, although the relationship between NNS consumption and weight reduction has been heavily researched [10,11,13], studies have not reached conclusive results on the relationship between NNS consumption and weight reduction. Interestingly, the present findings did not show a significant difference in body weight and BMI changes between the three groups over the 6-month intervention. As this study may not have been adequately powered to assess this relationship, future studies are needed to assess the relationship between SSB consumption reduction, NNS as a tool to do so, and potential changes in weight.

Data for this analysis were used from Talking Health, a 6-month intervention seeking to decrease participants’ consumption of SSB. A requirement for a participant’s enrollment was the consumption of at least 200 kcal/day of SSB [30]. Thus, by design, participants had a higher intake of SSB; consequently, their familiarity with or consumption of NNS might have been different than consumers who consumed less SSB. However, this level of SSB intake is comparable to SSB intake within rural populations [37]. Although, the generalizability of the results to other populations may be a limitation of this study. While this is a concern, research on SSB consumption in rural areas is important as residents in these areas are likely to consume more SSB than in other areas [37]. An additional limitation is the inherent issues related to self-reported dietary intake [38]. To account for this, interviewers were supervised by a PhD-level Registered Dietitian Nutritionist and the United States Department of Agriculture’s multiple-pass method was utilized to reduce misreporting issues [39]. Finally, this study only focused on the four reported NNS, rather than all eight currently approved by the U.S. Food and Drug Administration (FDA) or GRAS, as the version of NDS-R used at the time of the study did not provide information for all eight types. However, NDS-R, which was used to analyze the nutrient content of the dietary recalls, is state-of-the-art nutritional analysis software, which provides the most accurate NNS information available. 

Despite its limitations, this study has several strengths. These results show that many other dietary sources of NNS, in addition to diet sodas, are being consumed. The inclusion of other dietary sources helps ensure NNS intake is accurately measured and characterized. Likewise, this study focuses on various types of NNS. Our results showed that various types of NNS are found in different dietary sources, some sources including various types of NNS, and these are consumed in varying amounts. Accounting for different types of NNS, and the varied dietary sources they may be found in, can create a more accurate picture of NNS consumers and their intake patterns. 

## 5. Conclusions

With the consumption of added sugars on the rise, it is important to study alternative options that are appealing to consumers. Products sweetened with NNS are a potential alternative, but research on the effects of NNS on humans is limited. This study further supports the need to diversify the way we identify and quantify NNS consumption both via sources and types of NNS. Before research is able to accurately address the potential effects of NNS on health, it is necessary to improve study methodology to accurately characterize and assess NNS intake. Specifically, diet soda should not be used as a proxy for NNS consumption. This practice can incorrectly identify approximately 30% of NNS consumers as non-consumers. Furthermore, specific types of NNS should be examined, rather than total NNS, due to differences in metabolic pathways and dietary sources of NNS. These limitations are further compounded by the majority of some NNS types being primarily found in dietary sources other than diet sodas.

## Figures and Tables

**Table 1:** Changes in frequencies of non-nutritive sweetener (NNS) consumers and non-consumers in SIPsmartER participants over the 6-month sugar-sweetened beverage reduction intervention.

Baseline		6 Months			
NNS Consumers *n* (%)	Non-Consumers *n* (%)	Became NNS Consumers *n* (%)	Remained NNS Consumers *n* (%)	Became Non-Consumers *n* (%)	Remained Non-Consumers *n* (%)
30 (30)	71 (70)	25 (25)	21 (21)	9 (9)	46 (45)

NNS: non-nutritive sweeteners.

**Table 2:** Changes in mean daily non-nutritive sweetener (NNS) mg consumption in SIPsmartER participants by NNS type and total NNS over a 6-month sugar-sweetened beverage reduction intervention.

NNS Type	Baseline Mean ± SD	6 Months Mean ± SD	Mean difference ± SE ^a^
Aspartame (mg)	46.6 ± 107.5	83.8 ± 158.7	37.2 ± 13.9 **
Saccharin (mg)	0.6 ± 5.1	5.8 ± 32.9	5.2 ± 3.1
Sucralose (mg)	5.0 ± 21.8	26.7 ± 108.7	21.6 ± 11.1
Acesulfame Potassium (mg)	8.1 ± 26.9	7.8 ± 22.1	−0.3 ± 2.9
Total NNS (mg)	60.3 ± 127.3	124.1 ± 201.6	63.7 ± 18.5 ***

^a^ Paired samples *t*-test; ** *p* ≤ 0.01; *** *p* ≤ 0.001; NNS: non-nutritive sweeteners; SD: standard deviation; SE: standard error.

**Table 3:** Changes in the contribution of dietary sources to specific total daily non-nutritive sweetener (NNS) intake among SIPsmartER NNS consumers at baseline (*n* = 30) and 6 months (*n* = 46) ^a^.

Dietary Sources of NNS	Aspartame		Acesulfame Potassium		Sucralose		Saccharin	
	Baseline mg (%)	6 Months mg (%)	Baseline mg (%)	6 Months mg (%)	Baseline mg (%)	6 Months mg (%)	Baseline mg (%)	6 Months mg (%)
Diet soda	3563 (76)	7671 (91)	291 (36)	593 (76)	160 (31)	168 (6)	15 (23)	0 (0)
Diet tea	596 (13)	77 (1)	288 (36)	22 (3)	0 (0)	0 (0)	0 (0)	0 (0)
Juice or flavored drinks	208 (4)	242 (3)	223 (28)	132 (17)	206 (40)	70 (3)	0 (0)	0 (0)
Yogurt	124 (3)	62 (1)	0 (0)	0 (0)	114 (22)	0 (0)	0 (0)	0 (0)
Tabletop sweetener	140 (3)	176 (2)	0 (0)	0 (0)	0 (0)	2419 (90)	49 (77)	586 (100)
Cereal	50 (1)	0 (0)	0 (0)	0 (0)	0 (0)	0 (0)	0 (0)	0 (0)
Popcorn	0 (0)	0 (0)	0 (0)	0 (0)	22 (4)	0 (0)	0 (0)	0 (0)
Coffee cream substitute	0 (0)	0 (0)	8 (1)	6 (1)	8 (2)	6 (0)	0 (0)	0 (0)
Ice cream	17 (0)	85 (1)	0 (0)	0 (0)	0 (0)	0 (0)	0 (0)	0 (0)
Hot cocoa mix	0 (0)	146 (2)	0 (0)	0 (0)	0 (0)	0 (0)	0 (0)	0 (0)
Meal replacement product	0 (0)	0 (0)	0 (0)	26 (3)	0 (0)	26 (1)	0 (0)	0 (0)
Total (mg)	4698	8459	810	779	510	2689	64	586

^a^ Percentages may not total to 100 due to rounding; NNS: non-nutritive sweeteners.

**Table 4:** Demographic characteristics of SSB-NNS consumption change groups from SIPsmartER participants (*n* = 101) at 6 months ^a^.

Characteristics	Group 1: Decreased SSB with Increased NNS Consumption	Group 2: Decreased SSB but no Increase in NNS Consumption	Group 3: Increased or no Change in SSB, Regardless of Change in NNS Consumption
	(*n* = 36)	(*n* = 43)	(*n* = 22)
	*n* (%)	*n* (%)	*n* (%)
Sex *			
Male	7 (19)	11 (26)	0 (0)
Female	29 (81)	32 (74)	22 (100)
Mean age ± SD (years)	44.3 ± 12.9	43.3 ± 13.0	43.7 ± 13.2
Race/Ethnicity			
White	34 (94)	42 (98)	21 (96)
African American	2 (6)	1 (2)	1 (4)
Mean weight ± SD (kg)	94.8 ± 28.9	87.7 ± 21.5	93.1 ± 25.5
Mean body mass index (BMI) ± SD (kg/m^2^)	34.53 ± 9.1	31.68 ± 7.8	35.1 ± 9.5
BMI category			
Underweight (<18.5)	0 (0)	0 (0)	0 (0)
Normal (18.5–24.9)	4 (11)	11 (25)	2 (9)
Overweight (25–29.9)	10 (28)	8 (19)	5 (23)
Obese (≥30)	22 (61)	24 (56)	15 (68)
Education level			
High school graduate or less	12 (33)	14 (33)	8 (36)
Some college or more	24 (67)	29 (67)	14 (64)
Mean household income ± SD ($)	21,528 ± 16,347	23,547 ± 16,158	22,045 ± 16,432
Household income level ($)			
≤14,999	18 (50)	16 (37)	10 (45)
15,000–34,999	10 (28)	16 (37)	8 (36)
35,000–39,999	0 (0)	3 (7)	0 (0)
40,000–54,999	6 (17)	2 (5)	2 (9)
>55,000	2 (6)	6 (14)	2 (9)

SSB: Sugar-sweetened beverages; NNS: non-nutritive sweeteners; SD: standard deviation; BMI: body mass index; ^a^ X^2^ with adjusted standardized residuals for categorical variables; one-way ANOVA with post-hoc Tukey’s test for continuous variables; * *p* ≤ 0.05, participants in group 3 had significantly fewer males as compared to groups 1 and 2.

**Table 5:** Differences in mean daily sugar-sweetened beverage (SSB) and non-nutritive sweetener (NNS) consumption, body weight, and body mass index between SSB-NNS consumption change groups over time.

Characteristic	Group 1: Decreased SSB with Increased NNS Consumption (*n* = 36)			Group 2: Decreased SSB but no Increase in NNS Consumption (*n* = 43)			Group 3: Increased or no change in SSB, Regardless of Change in NNS (*n* = 22)			Significance Between Groups ^b^
	Baseline Mean ± SD	6 Months Mean ± SD	Mean Difference ± Std. Error ^a^	Baseline Mean ± SD	6 Months Mean ± SD	Mean difference ± Std. Error ^a^	Baseline Mean ± SD	6 Months Mean ± SD	Mean Difference ± Std. Error ^a^	
SSB (fl oz)	34.4 ± 23.5	7.3 ± 10.7	−27.0 ± 3.4 ***	37.1 ± 31.9	14.5 ± 13.9	−22.5 ± 4.0 ***	12.7 ± 11.8	20.2 ± 15.7	7.5 ± 1.5 ***	*F* = 19.419 *p* = < 0.001 ^c^
Total NNS (mg)	63.0 ± 129.9	255.4 ± 216.8	192.3 ± 29.2 ***^1^	13.2 ± 36.1	0.0 ± 0.0	−13.2 ± 5.5 *^2^	147.9 ± 184.0	151.6 ± 230.9	3.5 ± 55.0 ^1^	*F* = 17.953 *p* = < 0.001
Weight (kg)	94.2 ± 28.9	94.8 ± 28.8	0.5 ± 0.4 ^1^	89.0 ± 21.1	87.7 ± 21.5	−1.3 ± 0.8 ^1^	94.0 ± 25.7	93.0 ± 25.5	−1.0 ± 0.5 ^1^	*F* = 2.094 *p* = 0.129
BMI (kg/m^2^)	34.5 ± 9.0	34.5 ± 9.0	0.10 ± 0.2 ^1^	32.1 ± 7.8	31.6 ± 7.8	−0.51 ± 0.2 ^1^	35.45 ± 9.5	35.0 ± 9.5	−0.3 ± 0.2 ^1^	*F* = 1.620 *p* = 0.203

^a^ Significance determined by paired samples *t*-tests; ^b^ Significance determined by repeated measures ANOVAs using Tukey’s post hoc test, *p* ≤ 0.05; Labeled mean differences for each group with a common number across a row do not differ significantly; ^c^ Post hoc tests were unable to detect which mean values were significantly different between groups; SD: standard deviation; SSB: sugar-sweetened beverage; NNS: non-nutritive sweetener * *p* ≤ 0.05; *** *p* ≤ 0.001.
